# History of adaptation determines short‐term shifts in performance and community structure of hydrogen‐producing microbial communities degrading wheat straw

**DOI:** 10.1111/1751-7915.12678

**Published:** 2017-03-14

**Authors:** Idania Valdez‐Vazquez, Ana L. Morales, Ana E. Escalante

**Affiliations:** ^1^ Unidad Académica Juriquilla Instituto de Ingeniería Universidad Nacional Autónoma de México Blvd. Juriquilla 3001 C.P 76230 Querétaro Qro. Mexico; ^2^ Laboratorio Nacional de Ciencias de la Sostenibilidad Instituto de Ecología Universidad Nacional Autónoma de México Ciudad Universitaria Coyoacán C.P. 04510 Mexico City D.F Mexico

## Abstract

This study addresses the question of ecological interest for the determination of structure and diversity of microbial communities that degrade lignocellulosic biomasses to produce biofuels. Two microbial consortia with different history, native of wheat straw (NWS) and from a methanogenic digester (MD) fed with cow manure, were contrasted in terms of hydrogen performance, substrate disintegration and microbial diversity. NWS outperformed the hydrogen production rate of MD. Microscopic images revealed that NWS acted on the cuticle and epidermis, generating cellulose strands with high crystallinity, while MD degraded deeper layers, equally affecting all polysaccharides. The bacterial composition markedly differed according to the inocula origin. NWS almost solely comprised hydrogen producers of the phyla Firmicutes and Proteobacteria, with 38% members of *Enterococcus*. After hydrogen fermentation, NWS comprised 8% *Syntrophococcus*, an acetogen that cleaves aryl ethers of constituent groups on the aromatic components of lignin. Conversely, MD comprised thirteen phyla, primarily including Firmicutes with H_2_‐producing members, and Bacteroidetes with non‐H_2_‐producing members, which reduced the hydrogen performance. Overall, the results of this study provide clear evidence that the history of adaptation of NWS enhanced the hydrogen performance from untreated wheat straw. Further, native wheat straw communities have the potential to refine cellulose fibers and produce biofuels simultaneously.

## Introduction

Currently, the bioenergy market is a necessity for society. First‐generation (1G) ethanol production has assisted in the transition towards a low‐carbon economy; however, feedstocks used to produce it compete directly for resources with food production. In contrast, it has been suggested that second‐generation (2G) biofuels could be sustainable, as the use of lignocellulosic residual biomasses derived from forestry, agriculture and agroindustry does not compete directly with food production. Technological processes for 2G biofuel production should fulfil the commitments of white biotechnology achieving more efficient biomass degradation, consuming less energy and resources, generating less waste and obtaining suitable profits. The current technologies for 2G biofuels do not entirely fulfill these conditions, as these processes discharge effluents with residual chemicals, typically use elevated processing temperatures and pressures (> 100°C and 1 atm) and are not commercially viable (Rabemanolontsoa and Saka, 2016). To identify white biotechnologies in the biofuel market, several authors have tested microbial communities or consortia from different sources to mediate biological transformations from lignocellulosic feedstocks into biofuels. For example, microbial communities in anaerobic digesters simultaneously perform various tasks to transform complex substrates, generating methane‐rich biogases (Werner *et al*., 2011). Because microbial communities act in a single unit, the energy efficiency ratio of anaerobic digesters is higher than those bioprocesses with separate units dedicated to pretreatment, saccharification and fermentation (Börjesson and Mattiasson, 2007). Thus, the question remains whether a similar technology could be applied to produce another type of biofuel, such as bioalcohols or biohydrogen.

Hydrogen is a versatile, carbon‐free fuel. Either burning it directly in internal combustion engines or providing electrons for fuel cells, hydrogen supplies a source of pollution‐free energy. Dark fermentation is by far the biological method for producing renewable hydrogen that has the best opportunities for scaling up (Sen *et al*., 2016). New designs of 2G biorefineries now integrate H_2_‐producing fermenters as part of a strategy to enhance the end‐use energy efficiency (Sanchez *et al*., 2016). There are two routes to produce biohydrogen from lignocellulosic feedstocks: as mentioned earlier, configurations with separate units, or configurations using microbial consortia that integrate various operations (e.g. Valdez‐Vazquez *et al*., 2015; Sanchez *et al*., 2016).

There have been several reports on the performance of microbial consortia for hydrogen production from lignocellulosic biomasses. Typical microbial communities tested for this purpose include sludge from anaerobic digesters, composts and ruminal fluids (Chu *et al*., 2011; Pérez‐Rangel *et al*., 2015; Reginatto and Antônio, 2015). In addition, recent studies have highlighted the suitability of using microbial communities to produce liquid biofuels (Ho *et al*., 2011; Ronan *et al*., 2013). However, the drawback of using microbial communities has been the low product yields and extended conversion times. There are two potential explanations for the poor performance of such microbial communities. Microbial ecologists suggest that historical factors are determinant in shaping the functioning of native microbial communities (Martiny *et al*., 2006). For example, soil and sediment microbial communities perform better in their ‘native’ environment than when these communities are exposed to other new environmental conditions, suggesting the local adaptation of microbial communities to their original environments (Strickland *et al*., 2009; Reed and Martiny, 2013). A recent study showed that the microbial community naturally present in lignocellulosic biomasses outperformed other communities, such as anaerobic sludge, ruminal fluids and the communities present in soil to produce hydrogen from untreated wheat straw (Pérez‐Rangel *et al*., 2015). Members of the native microbial community were then isolated and used to integrate a synthetic microbial consortium for producing hydrogen from untreated wheat straw. The synthetic microbial consortium was unable to consume the entire fraction of the wheat straw xylan as the native microbial community did (Valdez‐Vazquez *et al*., 2015). Because in natural lignocellulosic biomasses, lignin forms stable lignin–carbohydrate complexes (Kajikawa *et al*., 2000), we assumed that still unknown members of the native wheat straw community are involved in the disintegration of the xylan–lignin complexes.

The starting point of this study is the design of a cellulosic biorefinery with two sequential bioprocesses, both mediated by microbial consortia. In the first stage, a microbial consortium acts on the lignocellulosic substrate only long enough to consume non‐cellulosic polysaccharides. The partially fermented substrate is then intended for producing bioalcohols (Valdez‐Vazquez *et al*., 2015). Keeping this in mind, in this study, we investigated two microbial communities of different origins, native of wheat straw (NWS) and from a methanogenic digester (MD), to characterize direct hydrogen production from wheat straw (WS), examine the microscopic arrangement to disintegrate lignocellulosic substrates and determine the composition of the communities using massive sequencing technologies. Overall, the three experimental approaches to characterize the microbial communities of interest enabled the elucidation of the relationship between the inocula origin (reflected as microbial diversity) and function, measured as the hydrogen production rate and substrate degradation of untreated wheat straw.

## Results and discussion

### Batch hydrogen production performance

**Figure 1:**
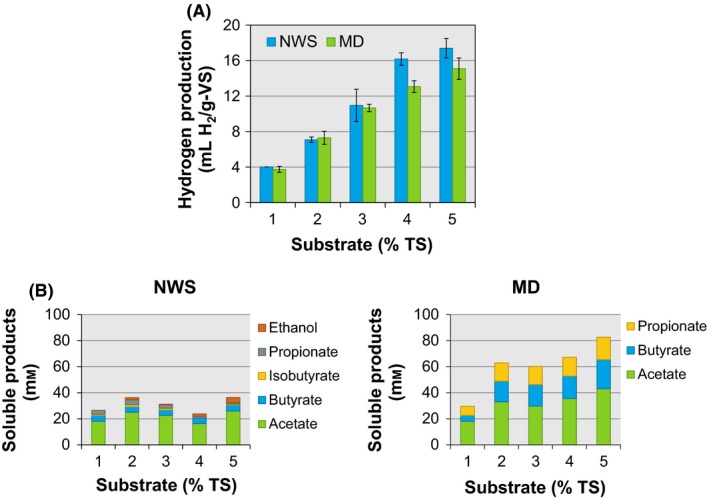
Performance of two microbial communities, native of wheat straw (NWS) and of a methanogenic digester (MD) for hydrogen production from untreated wheat straw after 7 days of incubation. A. Hydrogen production and B. soluble products. Errors bars indicate standard deviation (*n *=* *4).

The hydrogen production performance of the NWS community was compared with that from a MD using untreated WS as the sole carbon source (Fig. 1). Both microbial communities positively responded to the increase in the substrate concentration; NWS had an increasing in 3.3 ml of H_2_ with an increase of 1 g of total solids (TS) of substrate. In addition, NWS produced 1.15 times more hydrogen than the MD. In the range of substrate concentrations tested, neither of the two microbial communities exhibited substrate inhibition. Therefore, the substrate concentration at which hydrogen production reaches a maximum would be higher than 5% of TS for both populations. The profile of soluble products indicates that in NWS, already 70% of total soluble products comprised acetate (Fig. 1B). In contrast, MD produced on average 25% of the total soluble products as propionate, a metabolite whose production route represents a hydrogen sink (Reichardt *et al*., 2014). For both inocula, large quantities of acetate were recovered. The hydrogen/acetate ratio ranged from 0.5 to 1.5 for NWS and from 0.2 to 0.8 for MD. The hydrogen/acetate ratios suggest that acetogens were active in both microbial communities. Also, the metabolite profile in MD is a clear indicative of the existence of propionate producers competing for the substrate. A case study dealing with changes in hydrogen production with pH observed that the activity of propionate producers was stimulated at pH varying from 5.0 to 6.0. At this pH range, propionate was the major soluble metabolite positively related with the undermining of hydrogen production (Hwang *et al*., 2004). Given the initial pH of 6.5 in the batch tests, the activity of propionate producers could be stimulated. Besides propionate, the metabolite profiles for NWS and MD showed differences in the production of butyrate. MD produced quantities of butyrate that could have resulted in more hydrogen than NWS. However, the opposite was observed: NWS produced more hydrogen than MD. There could be at least one explanation for this behaviour, if, for example, butyrate‐producing pathways occurred in MD without hydrogen formation. Catabolism of fructose, a sugar present in the water‐soluble fraction of WS, produces butyrate without hydrogen (Falony *et al*., 2009; Tishler *et al*., 2015). Also, acetate – the main metabolite in the batch tests – acts as precursors of butyrate (Pryde *et al*., 2002). The results of microbial diversity revealed the identity of MD members whose metabolisms lead to the production of propionate and butyrate.

NWS was highly competitive in short‐term fermentations, with a hydrogen yield 1.15 times higher than that achieved with MD (17 ml of H_2_ per g of volatile solid (VS) for NWS versus 15 ml of H_2_/g‐VS for MD). After comparing the hydrogen performance under a mesophilic regime of different communities fermenting untreated wheat straw, the maximum yields reached by NWS and MD were at the same level than those previously reported ranging from 3 to 37 ml of H_2_/g‐VS (Chu *et al*., 2011; Quéméneur *et al*., 2012; Pérez‐Rangel *et al*., 2015). However, in terms of hydrogen production rate (R_m_, determined from the modified Gompertz equation), NWS displayed a higher value of *R*_m_ with 72 ml of H_2_/d than MD with a value of 55 ml of H_2_/d. NWS also outperformed the values of *R*_m_ reported for the other microbial communities which varying from 8 ml of H_2_/d to 68 ml of H_2_/d (Chu *et al*., 2011; Quéméneur *et al*., 2012; Pérez‐Rangel *et al*., 2015). In the present study, the increase in substrate concentration to nearly 5% TS resulted in an improvement in *R*_m_.

### Microscopic observations

**Figure 2:**
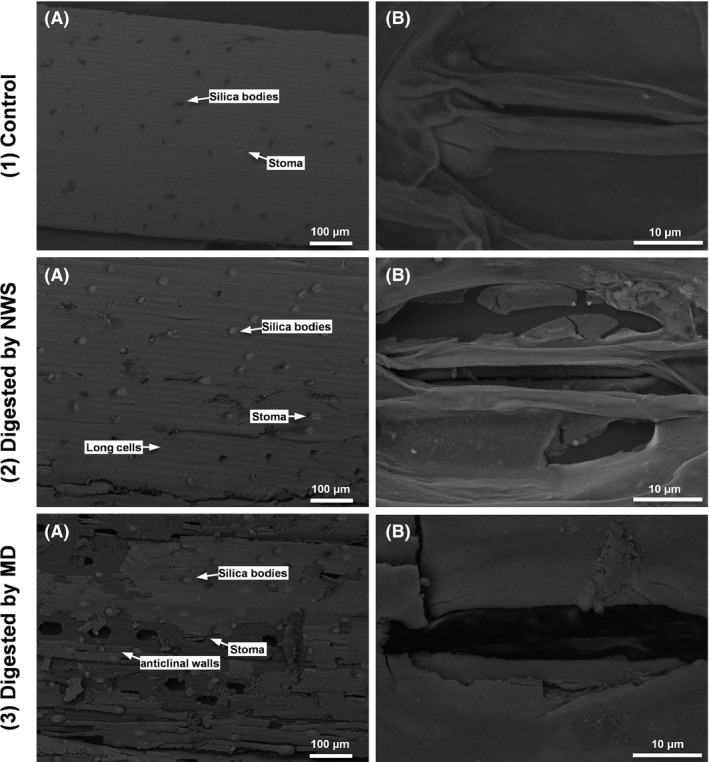
Scanning electron microscopy (SEM) photograph of wheat straw. A. Structure of the epidermis of wheat straw showing long cells, short cells with silica bodies and stomata. B. Structure of stomata from wheat straw. (1) Before hydrogen fermentation and after 7 days of hydrogen fermentation by (2) the native community of wheat straw (NWS) and (3) of a methanogenic digester (MD).

The total removal of volatile solids was similar for both microbial communities, showing on average 30% (± 3%) for NWS and 32% (± 9%) for MD. However, the extent degradation and functions of both hydrogen‐producing communities on the wheat straw differed. In Fig. 2, scanning electron microscopy (SEM) micrographs show the epidermal zone and stomata of WS. Unfermented WS had a smooth surface, reflecting the waxy layer (cuticle) that covers the epidermal cells (Xu, 2009). The epidermis containing long cells and short cells with silica bodies and stomata were observed, consistent with Andrade *et al*. (2012). All anatomical structures observed were intact (Fig. 2.1A,B). NWS superficially digested the WS, eliminating the cuticle and partially degrading the epidermis; thus, the tabular epidermal cells and silica bodies were exposed. Additionally, guard cells were partially degraded (Fig. 2.2A,B). In contrast, MD degraded deeper layers, and the epidermis, where stomata are localized, was peeled off, exposing the next layer, the cortex, which contains collenchyma and parenchyma cells (Xu, 2009). In SEM images for MD, guard cells of deeply degraded stomata and holes in place of stomata were common in the analysed surfaces. Moreover, the anticlinal walls [walls separating the two adjacent cells in the same layer of the epidermal cells (Jääskeläinen *et al*., 2013)] disappeared in some regions of the epidermis, presumably causing the structure of long cells to collapse (Fig. 2.3A,B).

Confocal laser scanning microscopy (CLSM) showed the site of polysaccharide degradation on these anatomical structures. Unfermented WS presented high green fluorescence with sinuous but well‐defined epidermal cells. The safranin staining of polysaccharides revealed irregular zones inside the epidermal cell anticlinal walls (arrowhead). These irregular zones play a relevant role in microbial degradation in NWS (Fig. 3.1A,B). After hydrogen fermentation, NWS left sinuous polysaccharide strands primarily corresponding to cellulose fibrils (Pérez‐Rangel *et al*., 2015). The activity of NWS was discontinuous, acting locally on irregular zones of the anticlinal walls and failing to penetrate deeper layers. Similar to the observation obtained from the SEM images, the stomatal guard cells were not extensively disturbed (Fig. 3.2A,B).

**Figure 3:**
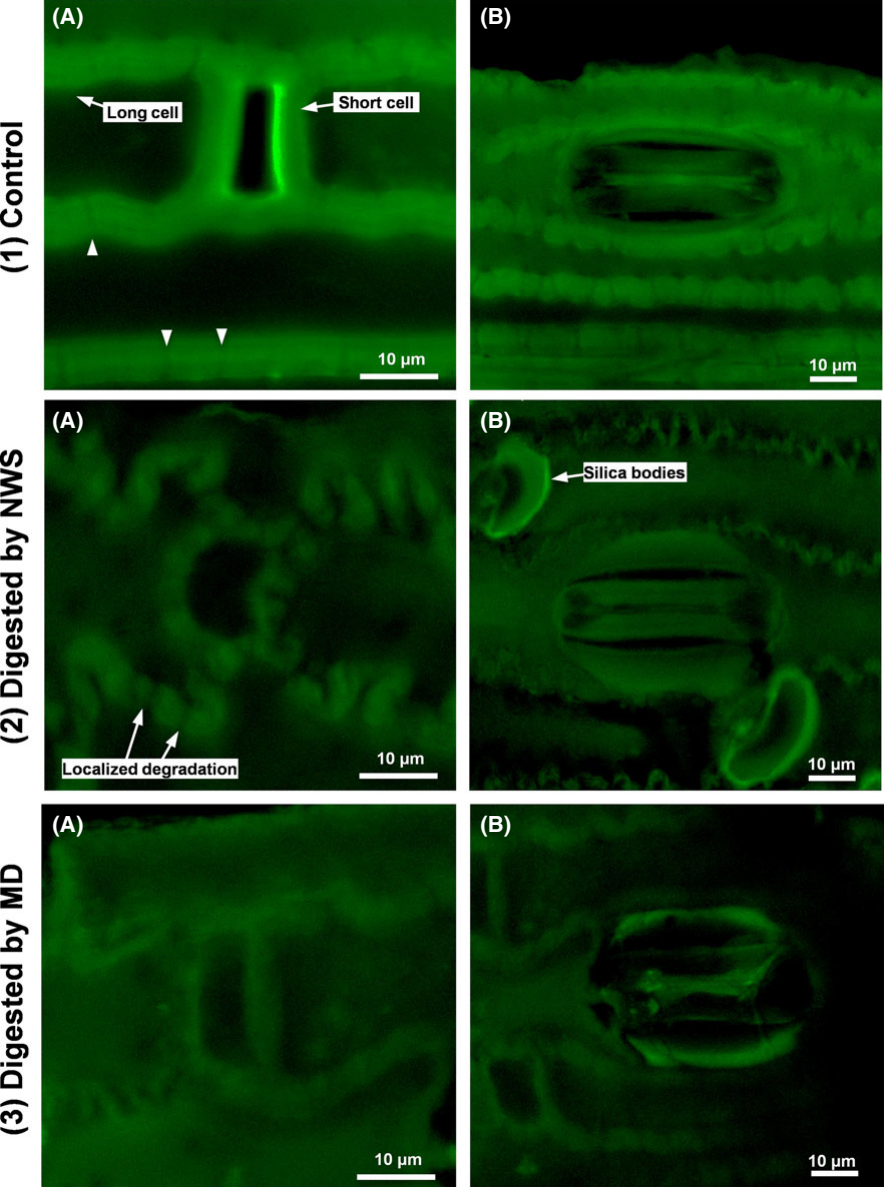
Confocal laser scanning microscopy (CLSM) images of polysaccharides in wheat straw stained with Safranine O (green fluorescence). A. Structure of the epidermis of wheat straw showing long and short cells. B. Structure of stomata and short cells with silica bodies. (1) Before hydrogen fermentation and after 7 days of hydrogen fermentation by (2) the native community of wheat straw (NWS) and (3) of a methanogenic digester (MD).

Regarding MD, the pattern of waviness of the epidermal cells was fairly conserved, and the uniform decrease in polysaccharide fluorescence indicated that MD showed a wide range of action on the carbohydrate fraction (Fig. 3.3A,B). The crystallinity index (CI) of cellulose after fermentation revealed that MD degraded equally amorphous and crystalline cellulose (CI of 50.1% for the unfermented WS versus 45.9% for the fermented WS). In contrast, NWS consumed amorphous cellulose leaving cellulose strands with high crystallinity (CI of 52.7% after fermentation, Fig. S1 in Supporting information).

### Bacterial diversity and composition

**Figure 4:**
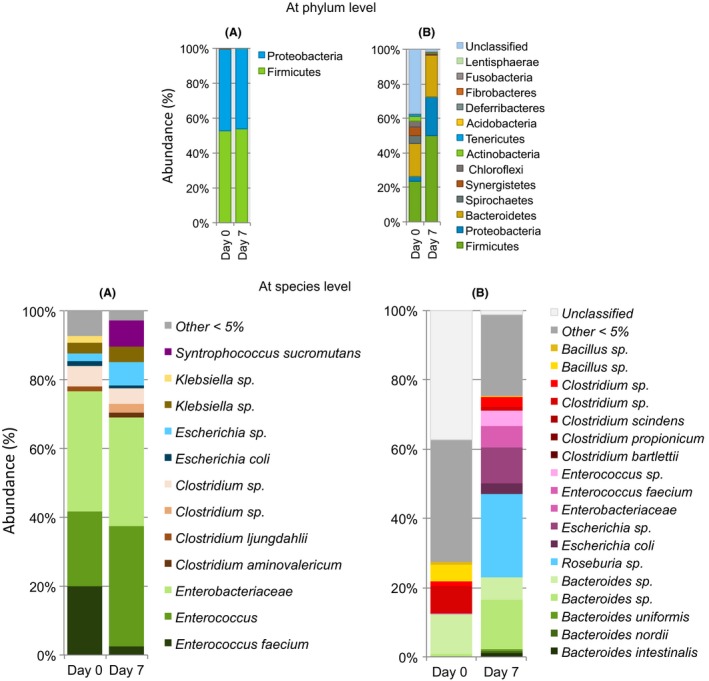
Proportion of major OTUs found in the H_2_‐producing reactors from wheat straw. A. Native wheat straw community and B. community of a methanogenic digester.

Original microbes of the studied communities and those remaining after 7 days of hydrogen fermentation were characterized using massive DNA pyrosequencing analysis (Fig. 4). A total of 49508 reads were obtained. After quality filters, NWS presented 9706 and 8870 reads before and after fermentation, respectively, while MD showed 9987 and 16220 reads before and after fermentation respectively. The median read length was 455 bps for all samples. Good‐quality reads were classified as different operational taxonomic units (OTUs) at the 97% sequence similarity cut‐off. The rarefaction analysis of OTUs showed a sufficient sampling effort for both microbial communities (see Fig. S2 in Supporting information). For NWS, more than 99.9% OTUs could be taxonomically assigned to at phylum level (Fig. 4). For MD, the percentages of taxonomically assigned OTUs were 63.7% and 98.9% before and after fermentation respectively (Fig. 4). The total number of distinct bacterial OTUs observed for NWS and MD were 21 and 75 respectively.

Members of Firmicutes and Proteobacteria evenly composed the original NWS (top panel in Fig. 4). The species richness observed for this microbial consortium was 19 with a Shannon index of 1.51. Notably, two species of *Enterococcus* dominated, composing 42% of the consortium. One member of the family Enterobacteriaceae represented 35% of the entire consortium. *Clostridium*, a typical hydrogen producer, represented only 7% of the original NWS including four different species (bottom panel in Fig. 4). Surprisingly, the population structure of NWS was nearly unaffected after hydrogen fermentation (species richness of 11 and Shannon index of 1.59). The two species of *Enterococcus* exhibited changes in abundance but together still accounted for almost 40% of total. The various species of *Clostridium, Escherichia* and *Klebsiella* also undergo minor changes. However, the most notable change was the increase in abundance of the genus *Syntrophococcus* from < 1% to 8%.

Original MD comprised 13 phyla, where Firmicutes and Bacteroidetes dominated, with 23.7% and 19.0% respectively (top panel in Fig. 4). The species richness for the original MD was 74 with a Shannon index of 3.09. In the original MD, *Clostridium* and *Bacteroides* represented 21.5% of the total of bacterial species. After hydrogen fermentation, the diversity and composition of the MD showed visible alterations (bottom panel in Fig. 4). The species richness decreased to 63 with a Shannon index of 2.43. At the end of fermentation, *Roseburia*,* Bacteroides* and *Escherichia* accounted for 60.2% of the total.

The bacterial composition greatly differed according to inocula origin. The most remarkable characteristic of the original NWS was the lack of aerobic members, which were expected based on previous reports of epiphytic bacteria, such as *Methylobacterium*,* Sphingomonas* and *Pseudomonas* (Vorholt, 2012). The only aerobic members observed in the original NWS that previously were reported as part of epiphytic communities were *Pseudomonas* and *Pantoea* (< 1%). Instead, the original NWS was enriched with facultative and strict anaerobes, likely reflecting the sun‐drying at which the wheat plants were subjected before harvest and the time in which the wheat straw was stored. Under indoor conditions (28°C and 55% relative humidity), all obligate aerobes died and only some genera, such as *Enterococcus,* survived on wheat straw. Previous reports have determined that *Enterococcus faecalis* survived well for extended periods under nutrient‐starvation conditions on solid substrates or water (Mackey and Hinton, 1990; Lebreton *et al*., 2014). These fortuitous events selected for bacterial species adapting to the new environmental conditions inside the anaerobic bioreactors. After hydrogen fermentation, *Enterococcus* and members of the family Enterobacteriaceae remained without major changes in abundance. The genus *Enterococcus* comprises members typically found in human and animal gastrointestinal tracts, the guts of insects, such as termites, plants, soil and water, and fermented foods and dairy products (Lebreton *et al*., 2014). Importantly, *Enterococcus* has been reported in a few hydrogen‐producing consortia (Liu *et al*., 2009; Pendyala *et al*., 2013). Recently, Valdez‐Vazquez *et al*. (2015) isolated and tested various strains of *Enterococcus* from the NWS. Such enterococcal strains efficiently convert soluble xylan. However, when cultivated under a natural polysaccharide matrix, these strains were incapable of completely degrading the xylan fraction consuming merely 30%. In natural lignocellulosic biomasses, xylan is linked to lignin via ether groups, forming xylan–lignin complexes (Kajikawa *et al*., 2000; Lawoko *et al*., 2006). The incapacity of *Enterococcus* to consume the entire xylan fraction could reflect the absence of some lignin‐releasing members from the NWS. After hydrogen fermentation, the abundance of obligate anaerobic, acetogen *Syntrophococcus*, increased. The genus *Syntrophococcus,* belonging to the family Lachnospiraceae, was originally isolated from rumen (Krumholz and Bryant, 1986). *Syntrophococcus* recognizes and cleaves the methyl groups within the polymeric structure of lignin as a one‐carbon source to release acetate and the corresponding hydroxyl derivatives. In anaerobic environments, this *O*‐demethylating acetogen plays a relevant role in the mineralization of ligno‐aromatic compounds in conjunction with other anaerobes that metabolize the aromatic ring structure (Doré and Bryant, 1990; Frazer, 1994; Bernard‐Vailhé *et al*., 1995). Because in native lignocellulosic substrates, xylan exists in the form of xylan–lignin complexes, *Syntrophococcus* twinned with fermentative bacteria could act as a catalyst for the degradation of the xylan fraction of native substrates, particularly in zones with highly lignified cells, such as the epidermal layer. In contrast, the cellulose fraction is not affected by the presence of these phenolic‐degrading acetogens. These observations support the findings of the present study, as *Syntrophococcus,* in conjunction with the remaining members of the NWS, primarily reported as H_2_ producers (*Enterococcus*,* Enterobacter*,* Clostridium*,* Klebsiella*,* Escherichia* and *Citrobacter*), hydrolysed the non‐cellulosic fraction of the WS to produce hydrogen, leaving unconsumed crystalline cellulose.

The original MD was distinguished based on the high level of diversity consistent with previous reports, where the phyla Firmicutes and Bacteroidetes were predominant (Klang *et al*., 2015). After hydrogen fermentation, five bacterial genera predominated: *Bacteroides, Roseburia, Escherichia, Enterococcus* and *Clostridium*. The genera *Escherichia, Enterococcus* and *Clostridium* represented the hydrogen‐producing population in the MD. Regarding *Roseburia*, some isolates express xylanase and endoglucanase activities producing H_2_, CO_2_ as well as formate, butyrate, succinate and lactate (Chassard *et al*., 2007, 2010). Similar to *Syntrophococcus*,* Roseburia* belongs to the family Lachnospiraceae, and these bacteria have been implicated in the disintegration of complex substrates. *Roseburia* along with other anaerobes belonging to the Clostridial clusters IV and XIVa are recognized as the main producers of butyrate by the microbial communities present in the human colon and rumen (Pryde *et al*., 2002). The catabolism of carbohydrates by *Roseburia* produces hydrogen. However, as outlined above, it seems that butyrate‐producing species were active without hydrogen formation. *Roseburia inulinivorans* perform the oligofructose degradation producing butyrate and CO_2_, but not H_2_ (Falony *et al*., 2009). *Roseburia intestinalis* converts acetate into butyrate with oligofructose as the sole energy source without hydrogen formation (Pryde *et al*., 2002). In this way, it appears that *Roseburia* representing almost 20% of the MD community could be responsible for butyrate formation, but with little or no contribution to the formation of hydrogen. Equivalent to one‐fifth of the abundance, the genus *Bacteroides*, belonging to the family Bacteroidaceae, was one of the most important bacteria in the MD. Some *Bacteroides* have been previously identified as relevant members of the fibrolytic microbial community in the human colon, degrading a wide range of polysaccharides, such as cellulose, xylan, starch and pectin generating acetate, propionate and succinate (Robert *et al*., 2007). In methane‐producing populations, *Bacteroides* plays a dual role, degrading complex polysaccharides and contributing to volatile organic acids, which are subsequently converted into methane, poorly contributing to hydrogen formation (Chassard *et al*., 2010). *Bacteroides* is primarily identified as a non‐H_2_‐producing cellulose‐degrading species. Therefore, in MD, *Bacteroides* could be responsible for decreasing the hydrogen yield when substrates without hydrogen formation were consumed to produce propionate. As mentioned earlier, an initial pH of 6.5 along with protein from the medium stimulated the activity of *Bacteroides* during the first days of incubation decreasing the potential of hydrogen production (Hwang *et al*., 2004; Walker *et al*., 2005).

At this point, it is important to stress that care is needed when comparing results of culture‐independent studies, such as the presented here, with the metabolic capabilities of isolated strains from the same genera or families. Some hypothesis from these comparisons can be derived and further investigated, like the role of specific species, genera or families in the overall functionality of bioreactors. To this end, it is in fact needed to continue strain isolation efforts to study directly strain metabolic capabilities, and also to pursue transcriptomic and proteomic efforts in bioreactors that will allow a better understanding of functionality.

### Biotechnological and ecological considerations

The findings of the present study have biotechnological and ecological implications. From a technological point of view, the members that integrate the NWS community are interesting to characterize because they have developed specialized enzymatic machinery to hydrolyse the xylan fraction with minor alterations in the cellulose fraction (refer to the microscopic observations). Thus, the partially refined cellulose can then be intended for producing other biofuels under a biorefinery approach. Two members are of special interest: *Enterococcus* and *Syntrophococcus,* both of which were highly abundant in NWS communities. *Enterococcus* is an H_2_‐producing, facultative anaerobe that survives during prolonged periods of starvation and produces bacteriocins (Leroy *et al*., 2003). These characteristics make *Enterococcus* highly desirable for large robust facilities for 2G biofuel production. For H_2_‐producing consortia enriched with *Enterococcus*, the anaerobic conditions for the regulation of growth could be relaxed, and the produced bacteriocins could limit the growth of undesired bacteria competing for the substrate. On the other hand, *Syntrophococcus* belongs to a family of acetogenic bacteria that catalyses *O*‐demethylation of constituent groups on the aromatic components of lignin. Other *O*‐demethylating bacteria also include *Acetobacterium woodi*,* Clostridium pfennigii*,* Eubacterium callandri* (Frazer, 1994). The specific cleavage of *β*‐aryl ethers bonds in lignin, accounting for approximately 50% of all the linkages in lignin, has been implicated in recovering valuable aromatic groups of lignin while refining cellulose (Reiter *et al*., 2013; Strassberger *et al*., 2013). To some extent, *Syntrophococcus* in conjunction with selected members of the NWS consortium could serve as an efficient biological pretreatment to refine cellulose fibers and to produce bioenergy in the form of hydrogen. Instead of dedicated units for high energy‐demanding pretreatments, saccharification and fermentation, 2G biofuel production facilities could install anaerobic fermenters with NWS microbial communities that, as shown in the present study, can degrade and efficiently ferment WS. The added environmental benefits are the elimination of acid or alkali compounds, elimination of pretreatment units obviates the formation of fermentation inhibitors, specific cleavage of ethers in lignin would preserve aromatic compounds for further industrial applications, and the refinement of cellulose strands for liquid biofuel production (ethanol/butanol). The implementation of such dual bioprocesses for biological pretreatment and hydrogen production would generate higher energy efficiency ratios and product yields than bioprocesses with separate units. In any case, additional studies using native strains of lignocellulosic biomasses are necessary to explore the roles of these bacteria in improving the rates of substrate consumption and product formation with other lignocellulosic substrates.

The results of the present study indicate that the inocula origin reflected differences in the microbial community composition, thereby affecting the hydrogen performance from untreated wheat straw. The primary population structure of NWS remained with minor alterations after hydrogen fermentations; only changes in abundances of some members were detected. In contrast, several members of MD become extinct after hydrogen fermentations. These results are a clear indication that NWS was already adapted to grow on WS. The time of storage for 6 months acted such as an adaptation process in which the microflora naturally present on the surface of WS could adapt to the lignocellulosic substrate before the hydrogen tests. In the course of these 6 months, major changes in the population structure of NWS could occur with the extinction of aerobic members and those with little resistance over long periods of storage. The long‐time storage resulted in a stabilized, adapted NWS consortium. These results resemble those microbial communities present in the stabilization period of fermentations or digestion processes. Microbial community dynamics of different bioprocesses involving lignocellulosic substrates indicate that microbial communities can be grouped into three categories based on pattern similarities: at the start‐up, during the growing period and at the stabilization period (Li *et al*., 2015; Yan *et al*., 2015). Also, the population structure remains stable over time if the operating conditions such as temperature and substrate are kept (Sun *et al*., 2015). By comparison with previous of microbial communities acting on lignocellulosic substrates, NWS can be considered such as a microbial community coming from the stabilization period of an adaptation process to WS, whereas MD consisted of a microbial community experiencing an adaptation process to the new substrate. Thus, history of adaptation of each microbial community determined the short‐term shifts in population structure.

Similar observations have previously been reported for other systems, such as soil and sediments, in terms of biogeochemical transformations and the activity of microbial communities (Strickland *et al*., 2009; Reed and Martiny, 2013). Studies of microbial ecology stated that historical factors determine the functioning of microbial communities, as these microbes behave better under their original environments (Strickland *et al*., 2009). In the present study, several unmeasured environmental conditions differed from the environment of which the microbial communities were derived; however, we assume that the substrate could be the main factor driving the behaviour of both consortia.

The above‐mentioned result highlights the importance of the ecological context and perspective, while selecting for specific microbially mediated enzymatic processes, such as lignocellulosic fermentation. Thus, the ecological perspective of these results raises questions about what microbial composition is better to disintegrate and ferment different lignocellulosic biomasses into target biofuels, where such microbial communities prosper, and how the bioreactor conditions affect the functioning of such communities. The microbial composition of NWS almost entirely included hydrogen producers and lignin‐degrading members that presumably acted together to increase xylan fermentation into hydrogen and soluble end‐products. In contrast, MD comprised non‐H_2_‐producing members that degraded substrates and therefore reduce the hydrogen yield. Based on these results, functionally robust H_2_‐producing communities must integrate members of Firmicutes and Proteobacteria, avoiding the sampling of natural communities in which Bacteroidetes is present.

In summary, this study presents the shifts in population structure during hydrogen production from a lignocellulosic substrate of two microbial communities with variations in life history traits. Differences between the NWS and MD communities were notable, before and after the hydrogen fermentations, where the former exhibited changes only in the proportion of their members, whereas the later had members that were extinct after fermentations. The history of the NWS community may have been decisive in shaping a better functioning than the MD community. Finally, new members in the NWS community were identified with potential for refining cellulose and producing hydrogen.

## Experimental procedures

### Lignocellulosic substrate and sources of inocula

Winter wheat straw (*Triticum aestivum* L.; cultivar. Urbina S2007) was mechanically harvested at 120 days after planting. The chopped straw samples were milled, and only particles retained in a 2 mm sieve were used in the present study. The chemical composition of wheat straw was previously reported by Valdez‐Vazquez *et al*. (2016), where the cellulose, hemicellulose and lignin contents were 387 g, 190 g and 173 g per kg respectively.

Methanogenic digester microbial community was obtained from the sludge of a digester fed with cow manure operated at a hydraulic retention time of 28 days, with a volatile solid removal efficiency of 35%, at pH 7.5, and a methane yield of 164 L‐CH_4_/kg‐VS. The sludge composition was 30 g l^−1^ of total solids (TS) and 22 g l^−1^ of volatile solids (VS).

Native wheat straw microbial communities were obtained inoculating the bioreactors with non‐sterile wheat straw. The wheat straw was stored indoors (28°C and 55% relative humidity) in opaque containers for 6 months prior to conducting the hydrogen production tests.

### Batch hydrogen fermentations

Serum bottles with volume of 150 ml served as anaerobic bioreactors. The bioreactors were run in quadruplicate for different wheat straw loads: 1, 2, 3, 4 and 5 g of wheat straw. For bioreactors with microbial communities from methanogenic digesters, the containers with wheat straw and medium were sterilized in an autoclave. After cooling, 20 ml of anaerobic sludge was added as inoculum (after filtering through gauze), and the bioreactors were supplemented with sterile medium to 100 g. The endogenous hydrogen production of MD was determined from bioreactors loaded with anaerobic sludge and medium without substrate (resulting in a negligible biogas accumulation in the whole incubation). For bioreactors with microbial communities from wheat straw, the bioreactors with non‐sterile wheat straw were supplemented with sterile medium to 100 g. The composition of the medium was 0.50 g of yeast extract, 0.25 g of tryptone, 0.25 g of meat extract, 2.10 g of MgSO_4_ and 2.00 g of KH_2_PO_4_, pH 6.5. All bioreactors were sealed with a rubber septum and a screw under an atmosphere of nitrogen and incubated at 37°C under static conditions.

### Analytical methods

Biogas accumulation was periodically measured as the displacement of a lubricated syringe. The biogas composition (H_2_, O_2_, CO_2_ and CH_4_) was analysed by gas chromatography following the analysis conditions previously reported (Cardeña *et al*., 2015). The soluble metabolites were measured from the culture supernatants after the samples were centrifuged at 8000 *× g* for 5 min and filtered through a 0.45 μm filter. Subsequently, the supernatants were analysed using a gas chromatograph (7890; Agilent Technologies, Palo Alto, CA, USA) equipped with a flame ionization detector and a 15 m long BD‐FFAP column (530 × 1 μm). The injector and detector temperatures were maintained at 190 and 210°C respectively. The temperature of the column was maintained at 60°C, after which it increased to 90°C at a rate of 15°C min^−1^ and then to 170°C at a rate of 25°C min^−1^ for 4 min. The carrier gas was nitrogen at 2.5 ml min^−1^. Total volatile solids and pH were determined according to APHA Standard Methods (Eaton and Franson, 2005).

### Microscopic analyses

SEM and CLSM were used to elucidate the mode of substrate disintegration of each microbial community on the wheat straw. Only untreated WS and bioreactors with the best hydrogen performance were analysed. SEM analysis was conducted using a JEOL JSM‐7600F SEM (JEOL Ltd., Tokio, Japan) operated at 10 kV with a low‐angle backscattered electron detector. The samples were coated with gold. Polysaccharides were stained with safranin O and subsequently visualized under a CLSM as previously reported (Pérez‐Rangel *et al*., 2015).

### Cellulose crystallinity index

Changes in cellulose crystallinity resulting from fermentations by different microbial communities with the best performance were determined using X‐ray powder diffraction according to Segal *et al*. (1959). The data were collected using a Rigaku Ultima IV (Rigaku Americas, TX, USA) under analysis conditions according to Valdez‐Vazquez *et al*. (2015). Crystallinity indices were presented as percentages.

### DNA extraction, pyrosequencing and data processing

Ten grams of samples of homogenized inocula generated at the end of hydrogen fermentation (communities in bioreactors with the best hydrogen performance only) was stored at −70°C until further analysis. Genomic DNA was extracted from 0.5 g of samples containing both solid particles and supernatants using the Power Soil DNA Extraction Kit^®^ (Mo Bio Laboratories Inc., Carlsbad, CA, USA) according to the manufacturer's instructions. For all samples, the four replicates were combined into a single mixture (25 μl of each DNA extract). Subsequently, the DNA concentration was adjusted to 20 ng μl^−1^ and sent to the Research and Testing Laboratory, RTL (Lubbock, Texas, USA), for the amplification of the 16S rRNA gene and pyrosequencing. Amplification of the 16S rRNA gene V1‐V3 region was performed using primers 28F–519R (Dowd *et al*., 2008), and subsequent sequencing using a Roche 454 FLX performed at RTL. A denoising protocol was used to remove short sequences, singleton sequences and noisy reads following the USEARCH algorithm (Edgar, 2010). All chimeric sequences were removed using the UCHIME software executed in *de novo* mode (Edgar *et al*., 2011). Only sequences longer than 250 bp with a quality score higher than 30 were kept. OTU selection was performed using the UPARSE algorithm (Edgar, 2013). The USEARCH global alignment algorithm along with a python program was used to assign the taxonomic identity for each of the OTUs using a database derived from the NCBI database (http://www.ncbi.nlm.nih.gov) and maintained by RTL. These sequence data were deposited in the GenBank database under accession number SRP076729. The species richness was calculated using the number of OTUs found in each sample. The Shannon diversity index (H) was calculated using the relative abundance of each OTU in the total sum.

## Conflict of interest

None declared.

## Supporting information


**Fig. S1.** X‐ray diffraction spectra of wheat straw for calculating crystallinity index (CI).
**Fig. S2.** Rarefaction plot of species richness, subsampling from 500 to 20 000 reads.
