# Supplementary material for: History of adaptation determines short‐term shifts in performance and community structure of hydrogen‐producing microbial communities degrading wheat straw
Source: Microb Biotechnol. 2017 Mar 14;10(6):1569–80. doi: 10.1111/1751-7915.12678 (PMC5658582; doi:10.1111/1751-7915.12678)
Supplement: Supplementary file 1 — Fig. S1. X‐ray diffraction spectra of wheat straw for calculating crystallinity index (CI). Fig. S2. Rarefaction plot of species richness, subsampling from 500 to 20 000 reads. [file MBT2-10-1569-s001.docx]

Hydrogen production performance and microbial diversity of native wheat straw microbial communities

Idania Valdez-Vazquez, Ana L. Morales, Ana E. Escalante

**^Microbial Biotechnology^**

**Supporting Information**

**Figure S1.**

X-ray diffraction spectra of wheat straw for calculating crystallinity index (CI).

**Figure S2.**

Rarefaction plot of species richness, subsampling from 500 to 20000 reads.
